# Association of Eating Window With Mortality Among US Adults: Insights From a Nationally Representative Study

**DOI:** 10.1111/acel.70230

**Published:** 2025-09-13

**Authors:** Ziling Mao, Haley Grant, Stephen B. Kritchevsky, Anne B. Newman, Samaneh Farsijani

**Affiliations:** ^1^ Department of Epidemiology, School of Public Health University of Pittsburgh Pittsburgh Pennsylvania USA; ^2^ Center for Aging and Population Health University of Pittsburgh Pittsburgh Pennsylvania USA; ^3^ Department of Biostatistics, School of Public Health University of Pittsburgh Pittsburgh Pennsylvania USA; ^4^ Department of Internal Medicine, Section on Gerontology & Geriatric Medicine and the Sticht Center for Healthy Aging and Alzheimer's Prevention Wake Forest University School of Medicine Winston‐Salem North Carolina USA

**Keywords:** cardiometabolic aging, chrono‐diet, circadian nutrition, longevity, national dietary surveillance, population‐based cohort

## Abstract

Time‐based diets have gained popularity for their health benefits, but their effects on human longevity remain unclear, with most evidence from short‐term human trials and animal studies. We determined the associations between eating window and mortality among U.S. adults. We conducted a prospective cohort study using NHANES 2003–2018 data linked to mortality records through December 2019. The analytic sample included 33,052 adults (aged 20 and above) with two complete 24‐h dietary recalls collected at baseline. Eating window was defined as the time between first and last consumption of any food/beverage containing > 0 kcal within 24 h. We used survey‐weighted Cox regression with Restricted Cubic Splines (RCS) to model nonlinear associations, treating eating window as both a continuous and categorical variable (< 8.0–≥ 15.0 h/day). Models were adjusted for sociodemographic, lifestyle, health, and dietary factors. Subgroup analyses were conducted by age, sex, and race/ethnicity. Over a median follow‐up of 8 years, there were 4158 all‐cause, 1277 cardiovascular, and 989 cancer deaths. *RCS models* showed a U‐shaped association between eating window and mortality, with the lowest risk at ~11–12 h/day (*p* = 0.004). Shorter windows (≤ 8 h) were linked to ≥ 30% higher all‐cause mortality, especially in older adults, and > 50% higher cardiovascular mortality in older adults, men, and Whites. Longer eating window categories (≥ 15 h/day) were associated with 25% higher all‐cause mortality (95% CI: 1.01–1.55). Moderate eating windows (~11–12 h/day) are linked to the lowest mortality risk, with deviations associated with higher risk. Differences across demographic groups highlight the need for personalized guidance.

## Graphical abstract

We examined eating window and mortality among 33,052 U.S. adults from NHANES 2003–2018 with mortality linkage. A U‐shaped association was observed: moderate eating windows of ~11–12 h/day were linked to the lowest risk, while shorter windows (< 8 h/day) and longer windows (≥ 15 h/day) were associated with 30% and 25% higher all‐cause mortality, respectively. Risks were especially pronounced for cardiovascular mortality in older adults and White participants.

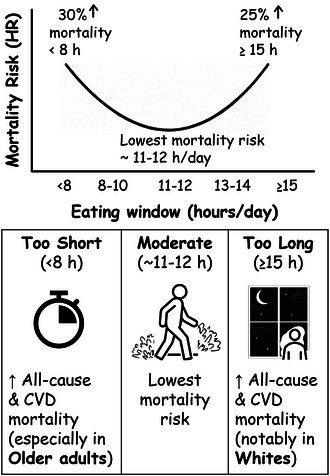

## 1 Introduction

Recently, time‐based dietary approaches, which emphasize consuming foods and beverages during specific daily time windows, have gained popularity due to their simplicity and flexibility (Varady et al. 2022). These diets may improve blood glucose levels, lipid profiles (Wilkinson et al. 2020; Lowe et al. 2020) and promote the production of beneficial metabolites like ketones (Duszka et al. 2020), potentially reducing oxidative stress (Mohr et al. 2021) and improving cardiometabolic health. Despite their popularity, most research consists of animal studies or short‐term human trials, with limited evidence on their associations with longevity. Additionally, the generalizability of these studies is limited by their small sample sizes and focus on specific groups, such as individuals with obesity, diabetes, or younger adults. This compromises the findings' broader applicability and representation across the life course. Given the scarcity of studies evaluating eating windows in relation to mortality in humans, largely due to the inherent challenges of conducting nutritional interventions over extended periods, there is a pressing need for large‐scale cohort studies across diverse demographics to provide critical insights into the associations between daily eating windows and longevity.

Despite data on differences in nutrient intake by age (Wakimoto and Block 2001) and sex (Bennett et al. 2018), variations in eating times across population subgroups remain underexplored. Our analyses of National Health and Nutrition Examination Survey (NHANES) data from 34,470 U.S. adults indicate that older adults (≥ 65 years) tend to eat breakfast earlier, consume their last meal earlier, and engage less frequently in late‐night eating (after 9 PM) compared to younger adults (Farsijani et al. 2023). Similarly, findings from our Study of Muscle, Mobility, and Aging (SOMMA) suggest that older adults may have slightly narrower eating windows on average, particularly among women and Black individuals (Mao et al. 2023). However, the broader impact of eating windows on mortality across diverse populations has not been extensively studied. Two recent NHANES‐based studies have suggested nonlinear associations between eating or fasting durations and mortality (Li et al. 2024; Zhang et al. 2024). However, these studies were either limited to specific age groups (e.g., adults aged ≥ 60 years (Zhang et al. 2024)) or relied on very broad eating window categories, potentially masking risks associated with very short eating windows (Li et al. 2024). Thus, the broader impact of eating window on various mortality outcomes across diverse population subgroups remains less well understood. Experimental work also supports a U‐shaped relation between the length of the daily eating window and key health outcomes. That is, very short daily eating windows can lead to insufficient energy or micronutrient intake, whereas very long windows favor late‐night eating, circadian misalignment, impaired glucose tolerance, and adverse lipid profiles (Wilkinson et al. 2020; Gill and Panda 2015; Manoogian et al. 2022). Together, these mechanistic and observational findings make nonlinear associations between eating window and mortality biologically plausible.

To address these significant gaps in knowledge, this study aimed to determine the associations between eating windows, derived from two 24‐h dietary recalls collected at baseline, and all‐cause, cardiovascular, and cancer mortality among US adults aged 20 years and above. We conducted stratified analyses by age, sex, and race/ethnicity to examine how the relationship between eating window and mortality varies across population subgroups, using nationally representative data from the NHANES collected between 2003 and 2018. We hypothesized that the relationship between eating window and mortality is non‐linear and potentially varying across population subgroups.

## 2 Methods

### 2.1 Study Population

The National Health and Nutrition Examination Survey (NHANES) employs a complex, multistage probability sampling method to conduct cross‐sectional surveys assessing the nutrition and health of the non‐institutionalized U.S. population (NHANES 2024). Approximately 10,000 nationally representative individuals are selected per 2‐year survey cycle for in‐home interviews and Mobile Examination Center (MEC) visits. The study protocols were approved by the Ethics Review Board of the National Center for Health Statistics (NCHS), and all participants provided written informed consent. In this study, we utilized publicly available data from the NHANES, which is de‐identified and accessible through the CDC website, and patients, or the public were not involved in the design, conduct, reporting, or dissemination plans of our research.

The cohort study analyzed data from eight NHANES cycles (2003–2004 through 2017–2018), starting with 70,451 participants who had available dietary data. We linked these data to the 2019 public‐use Linked Mortality File (LMF) for mortality. Exclusions were made for participants aged 19 and younger (*n* = 30,694), those with only one 24‐h food recall or with invalid 24‐h food recall (*n* = 5241), and those lacking identifying data for LMF linkage (*n* = 57). After further exclusions for extreme energy intakes (Frank et al. 2024; Lan et al. 2023) (< 600 or > 6500 kcal/d, *n* = 121), missing demographic data (*n* = 46), pregnancy (*n* = 646), missing (*n* = 388) or abnormal BMI (< 14 or ≥ 56 kg/m^2^, *n* = 164 (Flegal et al. 2019)), and missing covariates like smoking status and physical activity (*n* = 42), the final sample comprised 33,052 participants (Figure S1). Among all the adults aged 20 and above initially included in this study (*n* = 39,757), those who were excluded (*n* = 6705) were typically younger, predominantly male, and less likely to be White and married compared to those who were included (*n* = 33,052). Furthermore, excluded participants generally had lower incomes, were less physically active, and had a lower number of chronic conditions (Table S1).

### 2.2 Dietary Assessment

Detailed information on foods and beverages consumed, including the time of intake over a 24‐h period (midnight to midnight of the previous day), was collected by trained interviewers using the USDA's Automated Multiple Pass Method (AMPM) (NHANES 2015). AMPM is a validated five‐step interviewer‐administered protocol designed to systematically capture detailed information on the type, amount, and timing of all foods and beverages consumed over a 24‐h period, including commonly forgotten items such as snacks, thereby minimizing recall bias and under‐reporting. Each participant provided two 24‐h recalls at baseline in each NHANES cycle. Day 1 recall was collected via in‐person interviews at the MEC, with participants using measuring guides like cups, spoons, and rulers to report food quantities. Afterward, participants received measuring tools and a food model booklet with two‐dimensional representations to assist with the Day 2 recall, conducted via telephone 3–10 days later on a different weekday. The energy and nutrient content of foods were calculated using the USDA Food Surveys Nutrient Database. In the dietary recall interview, participants also detailed the clock times of their consumption of each food or beverage item. Using the clock‐time data recorded during step 3 of AMPM, we calculated the eating window (in hours per day) as the length of the time between the first and last food/beverage intake containing > 0 kcal energy consumed after midnight within a day (i.e., 00:00–23:59) (Farsijani et al. 2023). To estimate participants' usual intake, we calculated nutrient intake and eating window from 24‐h recalls for both Day 1 and 2. We then averaged the data from these recalls to reduce within‐person day‐to‐day variability in food and beverage consumption (Thompson and Subar 2013; Willett 2012a).

### 2.3 Mortality Ascertainment

Mortality data, including vital status and causes of death, were obtained from the 2019 NCHS LMF via linkage to the National Death Index (NCHS 2019). Deaths were categorized into 113 ICD‐10‐based categories and recorded into 9 major causes: heart disease, cancer, chronic lower respiratory disease, accidents, cerebrovascular disease, Alzheimer's, diabetes, influenza/pneumonia, and nephritis. The *primary* outcome was all‐cause mortality, and *secondary* outcomes were cardiovascular and cancer mortality. All‐cause mortality was based on the 2019 public‐use LMF “MORTSTAT” variable. Cardiovascular mortality included deaths from heart disease or cerebrovascular disease, and cancer mortality was defined as death from malignant neoplasms. Follow‐up time was calculated from the 24‐h recall interview to the date of death, censoring, or December 31, 2019. The median follow‐up was 8.1 years, with a maximum of 17.1 years. Due to the NHANES providing age in whole years, we used time since the MEC interview as the primary time scale for the time‐to‐event analysis.

### 2.4 Covariates

*Sociodemographic & lifestyle factors* were collected through self‐report, including age, sex, race/ethnicity, marital status, smoking, education level, and annual family income. Alcohol consumption was determined based on reported drinking frequency within the past 12 months. Physical activity was assessed using the Global Physical Activity Questionnaire. Minutes per week in moderate (3.0–6.0 METs) and vigorous (≥ 6.0 METs) activities were classified according to American Heart Association guidelines (Haskell et al. 2007), and MVPA was calculated by summing both. Participants self‐reported typical weekday sleep duration, with data available for NHANES cycles from 2005 to 2018 (Di et al. 2022). For employment status, occupational related variables in NHANES include “type of work done last week (OCD150)”, “usually work ≥ 35 hours/week (OCQ210)”, “main reason did not work last week (OCQ380)”, and “which best describes hours worked (OCQ265)”. Due to high levels of missingness in most variables (45%–85%), we selected “Type of work done last week”, which had minimal missing data (0.04%), as a surrogate for employment status and potential shift work. This variable indicates whether participants were working or not working (including those looking for a job).

#### 2.4.1 Anthropometrics

BMI (kg/m^2^) derived from measured weight (kg) and height (m), and categorized as underweight (< 18.5), normal (18.5–24.9), overweight (25.0–29.9), and obese (≥ 30.0). Fat and lean mass were assessed in a subset of participants (excluding 2007–2010 cycles) by dual energy X‐ray absorptiometry (Hologic QDR‐4500A, Bedford, MA, USA) (Kelly et al. 2009). Participants reported whether they attempted to lose weight in the past year (yes/no) and their perceived weight status as underweight, overweight, or about the right weight (Centers for Disease Control and Prevention (CDC) 2024).

#### 2.4.2 Dietary Related Covariates

Diet quality was evaluated using the 2015 Healthy Eating Index (HEI), where a higher score reflects a healthier diet (score: 0–100) (Krebs‐Smith et al. 2018). To address within‐person variation in dietary intake, we analyzed the days for the two dietary recalls, categorizing them as weekend/weekend, weekend/weekday, weekday/weekday, and weekday/weekend (Gicevic et al. 2021). Participants reported their experiences with poor appetite or overeating over the past two weeks, with response options ranging from “not at all” to “nearly every day”. This data was available for the 2005–2018 NHANES cycles (Ahluwalia et al. 2016). Participants reported their food security status, ranging from full to very low food security (Ahluwalia et al. 2016).

#### 2.4.3 Health Assessment

Participants self‐reported their general health as excellent, very good, good, fair, or poor. They also provided medical history, including 13 conditions: hypertension, hypercholesterolemia, diabetes, kidney disease, asthma, arthritis, heart failure, coronary heart disease, stroke, emphysema, bronchitis, liver disease, and cancer. A composite score (0–13) was created to quantify the number of chronic conditions reported (Ostrominski et al. 2023).

### 2.5 Statistical Analyses

Due to the complex sampling design of NHANES, all analyses incorporated sample weights, clustering, and stratification to be representative of the US adult population (Ahluwalia et al. 2016). Additionally, we accounted for this complex multistage stratified, clustered sampling design in our variance estimates using Taylor series linearization (Centers for Disease Control and Prevention 2024). Descriptive analyses were conducted to summarize the participant characteristics across categories of eating windows. These categories, < 8.00 h (Carlson et al. 2007; Sutton et al. 2018; Cienfuegos et al. 2020), 8.0–9.99 h (Lowe et al. 2020; Moro et al. 2016; Tinsley et al. 2019; Chow et al. 2020), 10.0–10.99 h (Wilkinson et al. 2020), 11.0–11.99 h (Gill and Panda 2015), 12.0–12.99 h (Farsijani et al. 2023; Jamshed et al. 2019), 13.0–14.99 h, and ≥ 15 h per day, were selected based on the most commonly used eating or fasting windows in time‐based diet trials in humans, their biological relevance to health outcomes, and our previous study characterizing the timing of intake among US adults in NHANES (Farsijani et al. 2023). Survey‐weighted ANOVA and survey‐weighted chi‐squared tests were used to compare the differences in continuous and categorical variables, respectively, across categories of eating window.

We assessed the relationship between eating window and mortality risk using survey‐weighted Cox regression to calculate hazard ratios (HRs) and 95% confidence intervals (CIs). We used Restricted Cubic Spline (RCS) regression, testing models with 3–7 knots and selecting the simplest model with an AIC within 4 of the lowest AIC (Harrell 2015). The optimal model, with knots at the 10th, 50th, and 90th percentiles, was chosen. Nonlinearity was evaluated by comparing linear and cubic spline models using a likelihood ratio test to detect nonlinear associations between daily eating window hours (i.e., treating eating window as a continuous variable) and mortality. All hazard models were adjusted for confounders selected a priori, along with additional covariates that showed significant differences across our eating window categories. *Model 1* adjusted for age, age‐squared (to account for the non‐linear relationship between age and mortality (Helmreich 2016)), sex, race/ethnicity, and BMI categories. *Model 2* included additional adjustments for smoking status, alcohol consumption, family income, total daily energy intake, diet quality, and the day of the week the dietary data was collected. *Model 3* was further adjusted for education, marital status, physical activity, self‐reported health status, number of chronic conditions, food security, self‐perceived weight status, and weight loss attempts.

We also assessed the relationship between our predefined eating window categories (i.e., treating eating window as a categorical variable) and mortality risk. This approach enabled us to examine nonlinear relationships without assuming a specific functional form for the relationship between eating window and mortality risk (Willett 2012b). The eating window of 12.0–12.99 h per day was used as the reference category as it represents the average eating window time in US adults from our prior NHANES study (Farsijani et al. 2023).

We conducted stratified analyses to assess the associations between eating window and mortality risk by age (< 65 or ≥ 65 years), sex (men or women), and race/ethnicity (White or Non‐White). Participants were grouped into young/middle‐aged adults (20–64 years) and older adults (≥ 65 years) due to the low mortality rate among younger individuals (140 events in 10,437) and middle‐aged adults (1011 events in 14,263). This consolidation enhanced clarity and highlighted contrasts with older adults, who had 3007 events in 8352 individuals. For race/ethnicity, small sample sizes necessitated combining the four categories (Hispanic, White, Black, and other/multiracial) into “White” and “Non‐White” to ensure reliable estimates. We performed several sensitivity analyses to test the robustness of our findings. Specifically, we repeated the fully adjusted models: (1) excluding deaths within the first year to reduce potential reverse‐causation bias, (2) restricting to adults aged ≥ 40 years to minimize the impact of deaths from external or non‐dietary causes (e.g., accidents and injuries), (3) additionally adjusting for sleep duration to address confounding by circadian factors, (4) additionally adjusting for employment status as a proxy for shift work, and (5) excluding participants with extreme eating windows (< 1st or > 99th percentiles) to test the influence of outliers. For all analyses, we included only complete cases, excluding records with missing data on any variables (Heymans and Twisk 2022). Exceptions were made for variables with some missing values: family income (1.2%), food security (1.6%), perceived weight status (0.3%), general health condition (4.8%), and alcohol consumption (17.9%). Missing values were imputed using mode substitution (Donders et al. 2006; Ambler et al. 2007), assigning the most common responses: family income (“≤ $34k”), food security (“full”), perceived weight status (“overweight”), general health (“good”), and alcohol consumption (“yes”). Analyses were performed using SAS version 9.4 (SAS Institute Inc.) and R version 4.3.1 for the RCS analysis. Two‐sided *p*‐values < 0.05 were considered statistically significant.

## 3 Results

### 3.1 Study Population

During a median follow‐up of 8.1 years (maximum 17.1 years), there were 4158 deaths (8.9% survey‐weighted), including 1277 (2.6%) cardiovascular and 989 (2.2%) cancer deaths. Table 1 presents the baseline characteristics of participants by eating window categories. Those with shorter eating windows were generally younger, female, Non‐Hispanic Black, unmarried, non‐smokers, had lower education and income levels, and reported fewer chronic conditions. They also had a higher prevalence of food insecurity, higher BMI, were less physically active, and had lower daily energy intake and diet quality compared to participants with longer eating windows.

**TABLE 1:** Characteristics of a national sample of 33,052 U.S. adults from NHANES (2003–2018) by eating window groups^a^.

Eating windows (h/d)	< 8.00	8.0–9.99	10.0–10.99	11.0–11.99	12.0–12.99	13.0–14.99	≥ 15.0	*p* ^b^
Participants, *n*	1473	4209	4308	6026	6204	7879	2953	
Age, years	41.2 ± 0.7	45.1 ± 0.5	47.5 ± 0.5	48.6 ± 0.4	48.4 ± 0.4	49.5 ± 0.3	45.3 ± 0.4	< 0.001
Age group, %^c^								< 0.001
Young/middle‐aged	1152 (85.7%)	3063 (80.4%)	3069 (78.3%)	4322 (78.8%)	4620 (80.7%)	5980 (81.9%)	2494 (88.5%)	
Older adults	321 (14.3%)	1146 (19.6%)	1239 (21.7%)	1704 (21.2%)	1584 (19.3%)	1899 (18.1%)	459 (11.5%)	
Women, %	753 (48.1%)	2369 (56.7%)	2484 (57.8%)	3319 (56%)	3239 (53.7%)	3678 (47%)	1172 (40.5%)	< 0.001
Race								< 0.001
Hispanics	356 (17.2%)	1216 (18.9%)	1167 (15.2%)	1580 (14.3%)	1472 (12.5%)	1679 (10.6%)	561 (10.9%)	
Non‐Hispanic White	427 (50.8%)	1546 (58.1%)	1819 (66%)	2705 (68.1%)	3015 (71.1%)	4086 (74.6%)	1458 (71.2%)	
Non‐Hispanic Black	567 (24.9%)	1086 (15.3%)	940 (12.1%)	1160 (9.7%)	1120 (9.3%)	1360 (8.1%)	657 (11.1%)	
Other/Multi‐Racial	123 (7.2%)	361 (7.6%)	382 (6.7%)	581 (7.9%)	597 (7.1%)	754 (6.6%)	277 (6.8%)	
Marital status, %								< 0.001
Married	681 (46.3%)	2320 (56.3%)	2536 (61.7%)	3757 (65.7%)	3966 (66.3%)	5106 (68.5%)	1713 (58.6%)	
Single again	330 (17.6%)	980 (18.4%)	1001 (18.4%)	1338 (17.8%)	1347 (18.5%)	1732 (18.1%)	639 (19.8%)	
Never married	462 (36%)	909 (25.2%)	771 (19.9%)	931 (16.5%)	891 (15.2%)	1041 (13.4%)	601 (21.6%)	
≥ College graduate, %	180 (14%)	691 (20.9%)	899 (25.2%)	1455 (31.5%)	1670 (34%)	2187 (35.3%)	684 (26.1%)	< 0.001
Income ≤ $34k, %	812 (45.4%)	2101 (42.5%)	1990 (37.7%)	2640 (32.6%)	2462 (30.2%)	3039 (28.7%)	1226 (34.3%)	< 0.001
Current employment status, %								< 0.001
No working/looking for a job	753 (43.3%)	2213 (45.2%)	2224 (44.7%)	2931 (41.3%)	2640 (36.8%)	3000 (32.3%)	954 (25.9%)	
Have job	719 (56.6%)	1993 (54.8%)	2082 (55.2%)	3092 (58.6%)	3561 (63.2%)	4879 (67.7%)	1997 (74.0%)	
Never‐smoked, %	821 (56.5%)	2503 (58.9%)	2515 (58.8%)	3477 (59.2%)	3450 (55%)	3993 (51.2%)	1325 (45.6%)	< 0.001
Alcohol drinking, %	1218 (85.4%)	3342 (83.8%)	3464 (84.3%)	4896 (85.2%)	5079 (85.3%)	6484 (85%)	2432 (84.9%)	< 0.001
Adult food security, %								< 0.001
Full	883 (65.5%)	2794 (73.1%)	3061 (76.6%)	4414 (80.2%)	4630 (81%)	5951 (81.8%)	2096 (77.3%)	
Marginal	214 (11.6%)	546 (10.4%)	489 (9.5%)	636 (7.7%)	599 (7.4%)	766 (7.2%)	331 (8.7%)	
Low food	206 (11.5%)	534 (9.9%)	448 (7.9%)	594 (7.2%)	581 (6.5%)	699 (6.4%)	288 (7.5%)	
Very low	170 (11.3%)	335 (6.6%)	310 (5.9%)	382 (4.8%)	394 (5.1%)	463 (4.5%)	238 (6.5%)	
Very good health status, %	293 (23.2%)	949 (27.6%)	1068 (33%)	1569 (33.6%)	1701 (32%)	2255 (34.1%)	787 (31.1%)	< 0.001
CVD, %	533 (29.4%)	1615 (32.5%)	1669 (32.7%)	2433 (35.7%)	2293 (31.9%)	3003 (33.5%)	1044 (30.4%)	0.006
Cancer, %	101 (5.8%)	366 (8.3%)	403 (9.0%)	512 (8.0%)	561 (8.7%)	703 (8.4%)	216 (6.7%)	0.043
Diabetes, %	211 (10.0%)	654 (11.5%)	723 (12.7%)	928 (11.6%)	888 (10.4%)	1147 (10.7%)	379 (10.4%)	0.120
Number of Chronic diseases, 0–13	1.23 ± 0.06	1.42 ± 0.04	1.5 ± 0.04	1.53 ± 0.03	1.41 ± 0.03	1.52 ± 0.03	1.42 ± 0.05	< 0.001
BMI (kg/m^2^), %								< 0.001
Underweight (< 18.5)	25 (1.6%)	72 (1.9%)	62 (1.8%)	77 (1.1%)	89 (1.4%)	113 (1.3%)	55 (1.9%)	
Normal (18.5–24.9)	362 (27.2%)	1056 (25.6%)	1102 (26.8%)	1657 (29.7%)	1733 (29.6%)	2161 (30.1%)	846 (29.6%)	
Overweight (25.0–29.9)	444 (28.4%)	1302 (30.3%)	1410 (31.9%)	1997 (32.5%)	2163 (34.6%)	2752 (34.2%)	997 (34.3%)	
Obese (≥ 30.0)	642 (42.8%)	1779 (42.2%)	1734 (39.5%)	2295 (36.7%)	2219 (34.5%)	2853 (34.4%)	1055 (34.2%)	
Lean mass, kg^d^	53.2 ± 0.7	51.1 ± 0.4	51.4 ± 0.4	51.1 ± 0.4	51.1 ± 0.3	52.7 ± 0.3	54.2 ± 0.5	< 0.001
Fat mass, kg^e^	29.4 ± 0.6	28.9 ± 0.4	29.2 ± 0.4	28.1 ± 0.4	27.7 ± 0.3	27.4 ± 0.3	26.9 ± 0.4	< 0.001
Sleep duration, h/d^f^	7.25 ± 0.06	7.26 ± 0.04	7.32 ± 0.03	7.30 ± 0.03	7.19 ± 0.03	7.01 ± 0.02	6.63 ± 0.05	< 0.001
MVPA, min/week	149.6 ± 10.0	161.2 ± 6.5	163.3 ± 8.8	169.5 ± 5.6	164.6 ± 6.2	185.8 ± 5.7	191.6 ± 8.5	< 0.001
Perceived weight status, %								0.003
Overweight	759 (53.2%)	2330 (58.1%)	2390 (56.9%)	3339 (57.1%)	3428 (57.6%)	4369 (56.5%)	1568 (53.3%)	
Underweight	73 (4.9%)	235 (4.9%)	213 (5%)	257 (3.8%)	293 (4.3%)	392 (4%)	189 (6.6%)	
About the right weight	641 (41.9%)	1644 (37%)	1705 (38.1%)	2430 (39.1%)	2483 (38.1%)	3118 (39.5%)	1196 (40.1%)	
Weight loss attempt (past year), %	475 (34.5%)	1332 (34.6%)	1442 (36.3%)	1970 (35%)	2026 (34.9%)	2482 (33%)	845 (31.1%)	0.224
Energy intake, kcal/d	1643 ± 27	1869 ± 16	1949 ± 19	2028 ± 15	2117 ± 15	2260 ± 16	2496 ± 27	< 0.001
Carbohydrate, %kcal/d	48.4 ± 0.3	49.0 ± 0.2	48.9 ± 0.2	48.6 ± 0.2	48.7 ± 0.2	48.7 ± 0.2	49.4 ± 0.3	< 0.001
Fat, %kcal/d	35.2 ± 0.3	34.7 ± 0.2	34.8 ± 0.2	34.9 ± 0.2	35.0 ± 0.1	35.1 ± 0.1	34.8 ± 0.2	< 0.001
Protein, %kcal/d	16.4 ± 0.2	16.4 ± 0.1	16.4 ± 0.1	16.5 ± 0.1	16.3 ± 0.1	16.2 ± 0.1	15.8 ± 0.1	< 0.001
Diet quality, 0–100	44.9 ± 0.4	48.2 ± 0.3	50.4 ± 0.3	52.3 ± 0.3	52.4 ± 0.3	52.6 ± 0.3	50.3 ± 0.4	< 0.001
Eating window, h/d	6.62 ± 0.04	9.1 ± 0.01	10.43 ± 0.01	11.42 ± 0.01	12.39 ± 0.01	13.74 ± 0.01	16.52 ± 0.05	< 0.001
Food recall days of the week, %								< 0.001
Weekday/weekday	589 (45.6%)	1637 (40.3%)	1828 (44.4%)	2561 (42.7%)	2770 (47.1%)	3887 (52.3%)	1483 (54.3%)	
Weekday/weekend	292 (24.6%)	810 (25.2%)	822 (25.2%)	1141 (26.6%)	1078 (24.1%)	1210 (21.1%)	425 (18.9%)	
Weekend/weekday	518 (23.9%)	1575 (27%)	1451 (22.8%)	2093 (24.4%)	2143 (23.3%)	2576 (22.9%)	952 (21.3%)	
Weekend/weekend	74 (5.9%)	187 (7.5%)	207 (7.5%)	231 (6.3%)	213 (5.4%)	206 (3.8%)	93 (5.6%)	
Appetite poor/overeating, %^g^								< 0.001
Not at all	943 (72.8%)	2854 (75.8%)	2941 (77.6%)	4184 (80.3%)	4397 (78.5%)	5426 (79.6%)	1912 (73.9%)	
Several days	193 (14.7%)	526 (14.9%)	553 (14.3%)	741 (13.2%)	748 (14.5%)	967 (14%)	401 (16%)	
More than half the days	75 (5.6%)	162 (4.7%)	172 (4.7%)	210 (3.7%)	209 (4.1%)	295 (3.5%)	144 (5.7%)	
Nearly every day	88 (6.9%)	178 (4.6%)	164 (3.4%)	210 (2.8%)	183 (3%)	255 (2.9%)	145 (4.4%)	

Abbreviations: BMI, body mass index; CVD, cardiovascular disease (including hypertension, heart failure, coronary heart disease, or stroke); MVPA, moderate to vigorous physical activity.

^a^ Survey‐weighted percentages and mean ± SE were estimated using US population weights. Statistics were calculated after missing imputation.

^b^ *p* values from survey‐weighted ANOVA for continuous variables and survey‐weighted chi‐squared test for categorical variables.

^c^ Age categories: Young and middle‐aged (20–64 years) and older (≥ 65 years) adults.

^d^ Lean mass (*n* = 15,556) assessed by DXA (QDR‐4500A, Hologic Inc., Bedford, MA, USA) which was not available for NHANES cycles 2007–2010.

^e^ Fat mass (*n* = 15,387) assessed by DXA (QDR‐4500A, Hologic Inc., Bedford, MA, USA) which was not available for NHANES cycles 2007–2010.

^f^ Self‐reported usual sleep duration on weekdays (*N* = 29,200) was not available for NHANES cycles 2003–04.

^g^ Appetite (*N* = 29,313): Assessed using the question ‘Over the last 2 weeks, how often have you been bothered by poor appetite or overeating?’ from NHANES cycles 2005–2018.

### 3.2 Eating Window and All‐Cause Mortality

**FIGURE 1:**
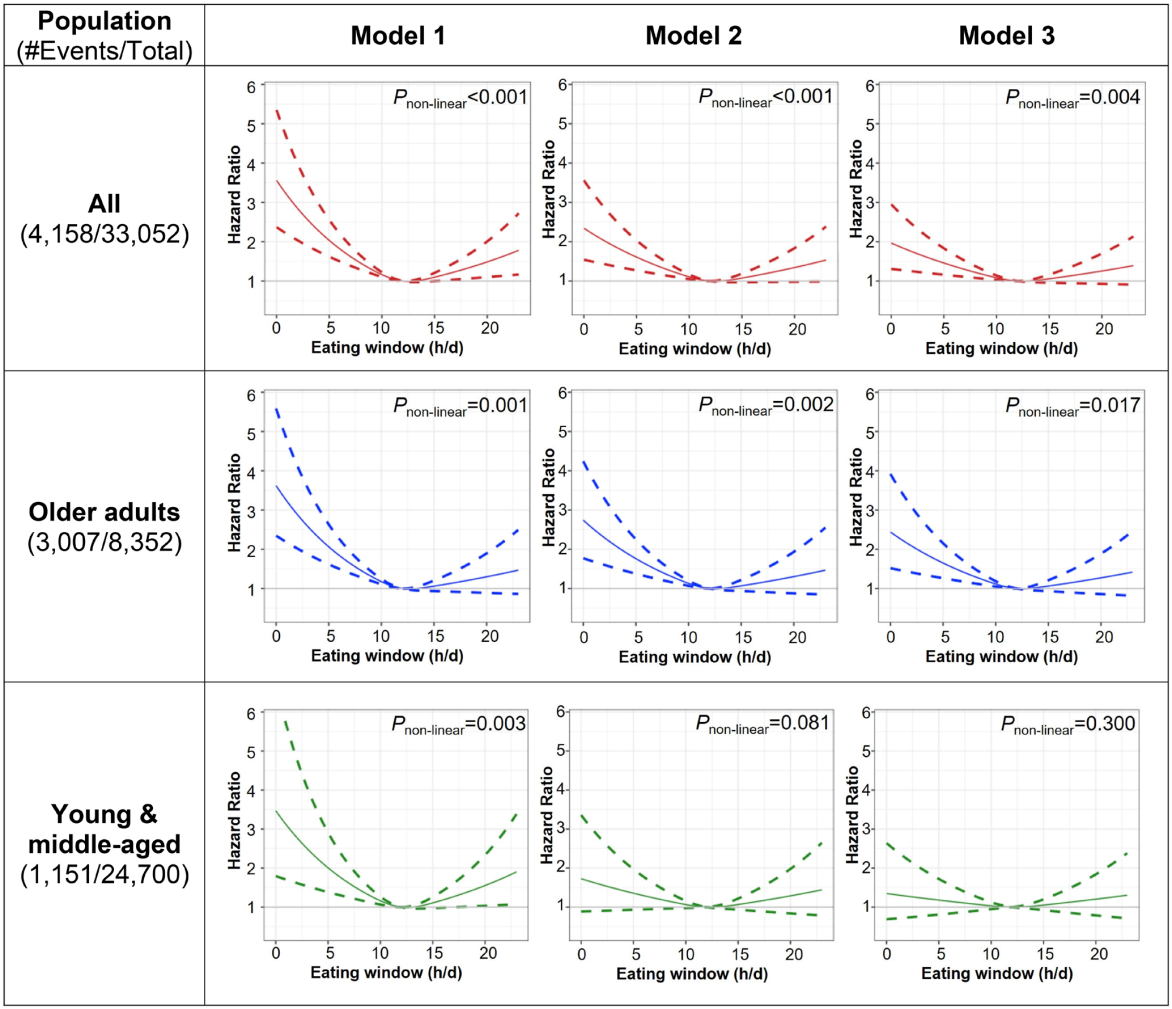
Survey‐Weighted Cox Regression with Restricted Cubic Splines (RCS) transformation for the association between eating window and all‐cause mortality among US adults (*N* = 33,052): Overall and by age groups (Young and middle‐aged (20–64 years, *n* = 24,700) and older (≥ 65 years, *n* = 8352) adults). Survey‐weighted multivariable Cox proportional hazards regression models were used to calculate hazard ratios (HRs; solid lines) and 95% confidence intervals (CIs; dashed lines). The shape of the associations was evaluated using RCS regression. Nonlinearity was assessed by comparing models: One with a linear term and another with cubic spline terms, using a likelihood ratio test to determine if there was a nonlinear relationship between the eating window and mortality. All hazard models were adjusted as follows: **Model 1**: Adjusted for age, age^2^, sex, race, and BMI categories. **Model 2**: Further adjusted for total calorie intake, diet quality, day of dietary intake, family income, alcohol intake, and smoking status. **Model 3**: Additionally adjusted for self‐reported health conditions, number of chronic conditions, moderate‐to‐vigorous physical activity, food security, self‐perceived body weight, attempts to lose weight in the past year, marital status, and education.

In the overall population, our RCS regression analysis showed a significantly non‐linear, U‐shaped relationship between the eating window (i.e., treating eating window as a continuous variable) and HRs for all‐cause mortality (Figure 1). The lowest risk was observed at an eating window of about 11–12 h per day in our multivariable adjusted Model 1, which controlled for age, age squared, sex, race/ethnicity, and BMI categories. Although further adjustments in Model 2 and in Model 3 slightly attenuated the association, the non‐linear relationship remained significant (*p* = 0.004, Figure 1). Specifically, shorter eating windows of 8 h or less per day were associated with a significantly higher risk of all‐cause mortality, with HR of 1.3 or higher in the fully adjusted model. Conversely, longer eating windows were associated with a higher mortality risk in our RCS Model 1, though the association did not remain statistically significant in the fully adjusted Model 3 as the lower confidence interval crosses the threshold of 1 (Figure 1).

**FIGURE 2:**
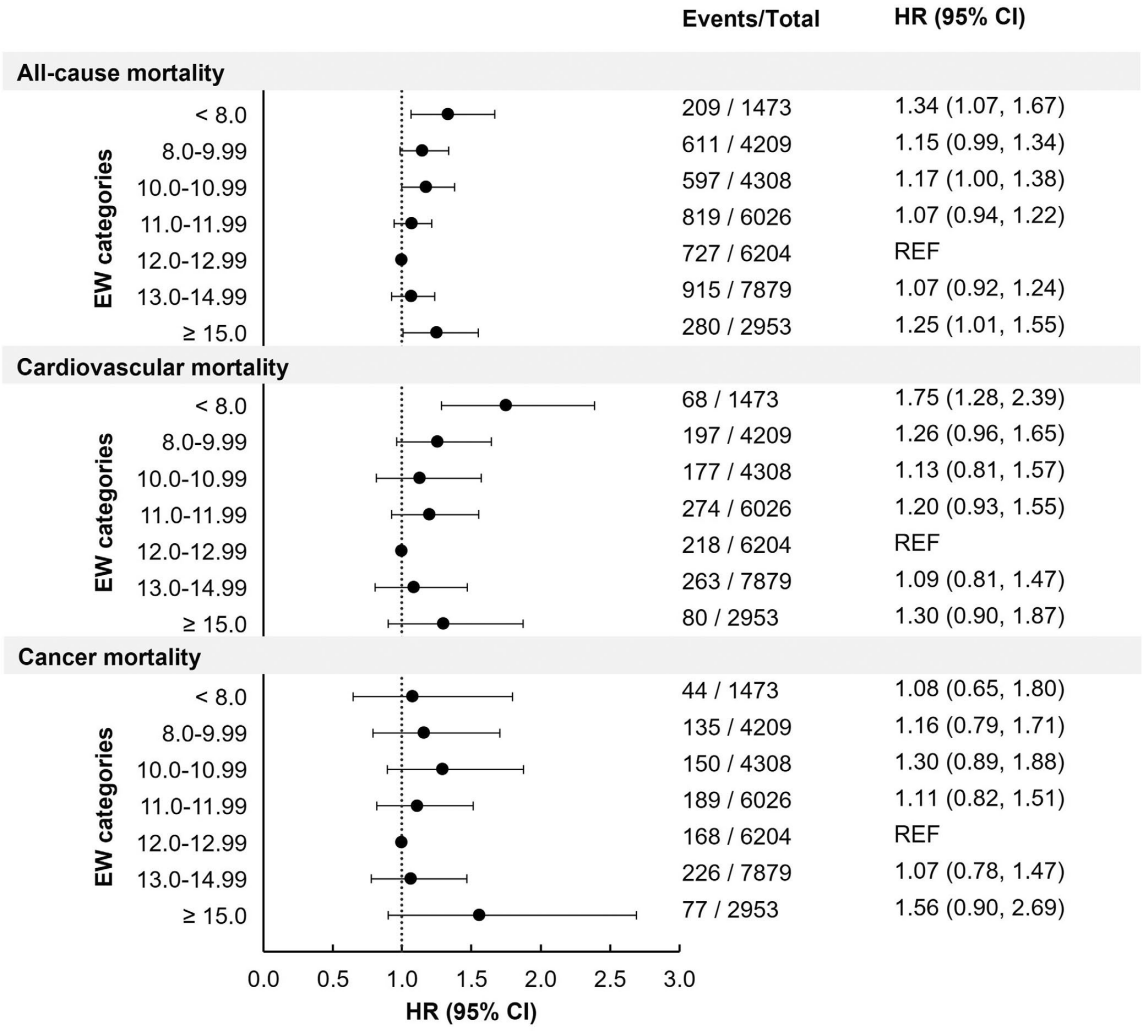
Associations between daily eating windows (EW) categories and all‐cause and cause‐specific mortality among U.S. adults aged ≥ 20 years (*N* = 33,052). Hazard ratios (HR) and 95% Confidence Intervals (CIs) obtained from survey‐weighted Cox regression model adjusted for age, age^2^, sex, race, and BMI categories, total calorie intake, diet quality, day of dietary intake, family income, alcohol intake, smoking status, self‐reported health conditions, number of chronic conditions, moderate‐to‐vigorous physical activity, food security, self‐perceived body weight, attempts to lose weight in the past year, marital status, and education.

In addition to our RCS analysis, we examined the relationship between all‐cause mortality and predefined eating window categories commonly used in time‐based diet trials in human studies to facilitate interpretation of the findings for practical application. These categories, ranging from short (< 8.00 h/day) to long (≥ 15 h/day) eating windows, revealed a U‐shaped relationship with all‐cause mortality risk in the overall population (Figure 2). In our fully adjusted Model 3, the eating window category of < 8.00 h/day was associated with a 34% greater mortality (HR: 1.34; 95% CI: 1.07–1.67; *p* = 0.012) compared to the 12.0–12.99 h/day reference category. Conversely, longer eating window categories, specifically ≥ 15.0 h/day, were associated with a 25% higher risk of all‐cause mortality (HR: 1.25; 95% CI: 1.01–1.55; *p* = 0.041), when compared to our reference category in the fully adjusted Model 3 (Figure 2).

To test the robustness of these findings, we performed additional sensitivity analyses. First, excluding participants who died within the first year of follow‐up, shorter eating window categories of < 8.00 h/day remained associated with a 27% higher mortality risk (HR: 1.27; 95% CI: 1.01–1.59; *p* = 0.043) (Table S2). Second, we excluded younger participants (aged 20–39 years) from the analysis, as their mortality (140 deaths among 10,437 participants) may be more influenced by external and non‐dietary factors, such as accidents or injuries. Limiting our analysis to participants aged 40 and above, we observed similar associations: shorter eating window categories of < 8.00 h/day (HR: 1.40; 95% CI: 1.12–1.75; *p* = 0.004) and 8.00–9.99 h/day (HR: 1.17; 95% CI: 1.01–1.36; *p* = 0.042), as well as longer eating window categories of ≥ 15.00 h/day (HR: 1.27; 95% CI: 1.03–1.58; *p* = 0.028), were associated with higher all‐cause mortality rates (Table S3). Third, further adjusting our models for sleep duration (available in NHANES cycles from 2005 to 2018) (Table S4) and employment status, which indirectly suggests whether individuals had regular or irregular work schedules (Table S5), did not alter the observed relationships between eating window categories and mortality risk. Finally, excluding extreme values of eating window (below the 1st percentile and above the 99th percentile, < 5 and > 20 h per day, respectively) had minimal impact on the observed associations with mortality, further supporting the robustness of our findings (Table S6).

We conducted stratified analyses to determine how age, sex, and race/ethnicity influence the relationship between eating window and mortality. In older adults, our fully adjusted RCS analysis showed a significant non‐linear association between eating window (treated as a continuous variable) and mortality, with the lowest observed risk associated with eating window of 11–12 h per day (*p* = 0.017) (Figure 1). Notably, shorter eating windows (≤ 8 h/day) were associated with a significantly higher risk of all‐cause mortality, with HR of 1.3 or higher and 95% confidence intervals consistently above 1. In contrast, longer eating window (beyond 15 h) showed an upward trend in HRs, though this did not reach statistical significance. In younger adults, a non‐linear U‐shaped relationship between eating windows and higher all‐cause mortality risk was observed in RCS Model 1 (*p* = 0.003). However, this association weakened in the fully adjusted Model 3 (*p* = 0.300), suggesting that after adjusting for additional covariates, neither shorter nor longer eating windows were significantly linked to mortality risk (Figure 1).

**FIGURE 3:**
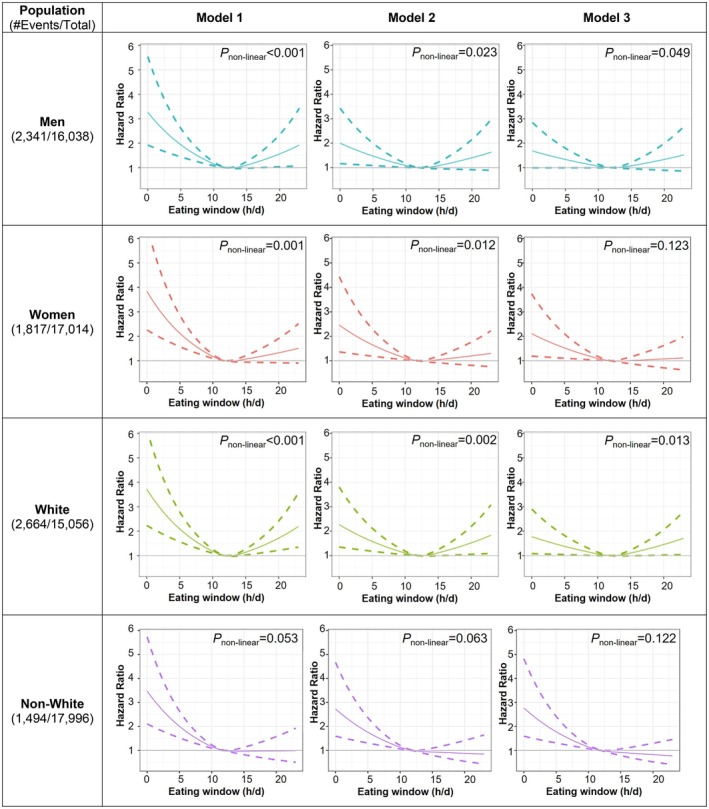
Survey‐Weighted Cox Regression with Restricted Cubic Splines (RCS) transformation for the association between eating window and all‐cause mortality among US adults (*N* = 33,052): Sex (Men (*n* = 16,038) or Women (*n* = 17,014)) and race/ethnicity (White (*n* = 15,056) or Non‐White (*n* = 17,996)) groups. Survey‐weighted multivariable Cox proportional hazards regression models were used to calculate hazard ratios (HRs; solid lines) and 95% confidence intervals (CIs; dashed lines). The shape of the associations was evaluated using RCS regression. Nonlinearity was assessed by comparing models: One with a linear term and another with cubic spline terms, using a likelihood ratio test to determine if there was a nonlinear relationship between the eating window and mortality. All hazard models were adjusted as follows: **Model 1**: Adjusted for age, age^2^, sex, race, and BMI categories. **Model 2**: Further adjusted for total calorie intake, diet quality, day of dietary intake, family income, alcohol intake, and smoking status. **Model 3**: Additionally adjusted for self‐reported health conditions, number of chronic conditions, moderate‐to‐vigorous physical activity, food security, self‐perceived body weight, attempts to lose weight in the past year, marital status, and education. Stratified variable was not adjusted for in corresponding sex‐ or race‐stratified models.

In our sex‐stratified RCS analysis, a non‐linear U‐shaped relationship between eating windows (treated as a continuous variable) and all‐cause mortality was observed in both men and women in Model 1 (*p* < 0.001). This relationship remained significant for men in Model 3 (*p* = 0.049) but not for women (*p* = 0.132; Figure 3). However, shorter eating windows were still linked to higher mortality in women, as the 95% confidence intervals remained above 1. In men, the association weakened in Model 3, with the confidence intervals slightly crossing 1, indicating a less significant trend. Longer eating windows were not linked to higher mortality in either sex.

Our race‐stratified RCS analysis revealed a significant U‐shaped relationship between eating window and mortality risk in White participants in the fully adjusted Model 3 (*p* = 0.013). In RCS analysis, when treating eating window as a continuous variable, eating windows < 8 h/day and > 17 h/day were associated with higher mortality risk (HR ≥ 1.2; Figure 3). In contrast, the U‐shaped pattern was less pronounced for Non‐Whites, where only shorter eating windows (≤ 8 h/day) were linked to significantly higher mortality risk (HR ≥ 1.4). No significant association was found between longer eating windows and mortality in Non‐Whites (Figure 3).

### 3.3 Cardiovascular Mortality

**FIGURE 4:**
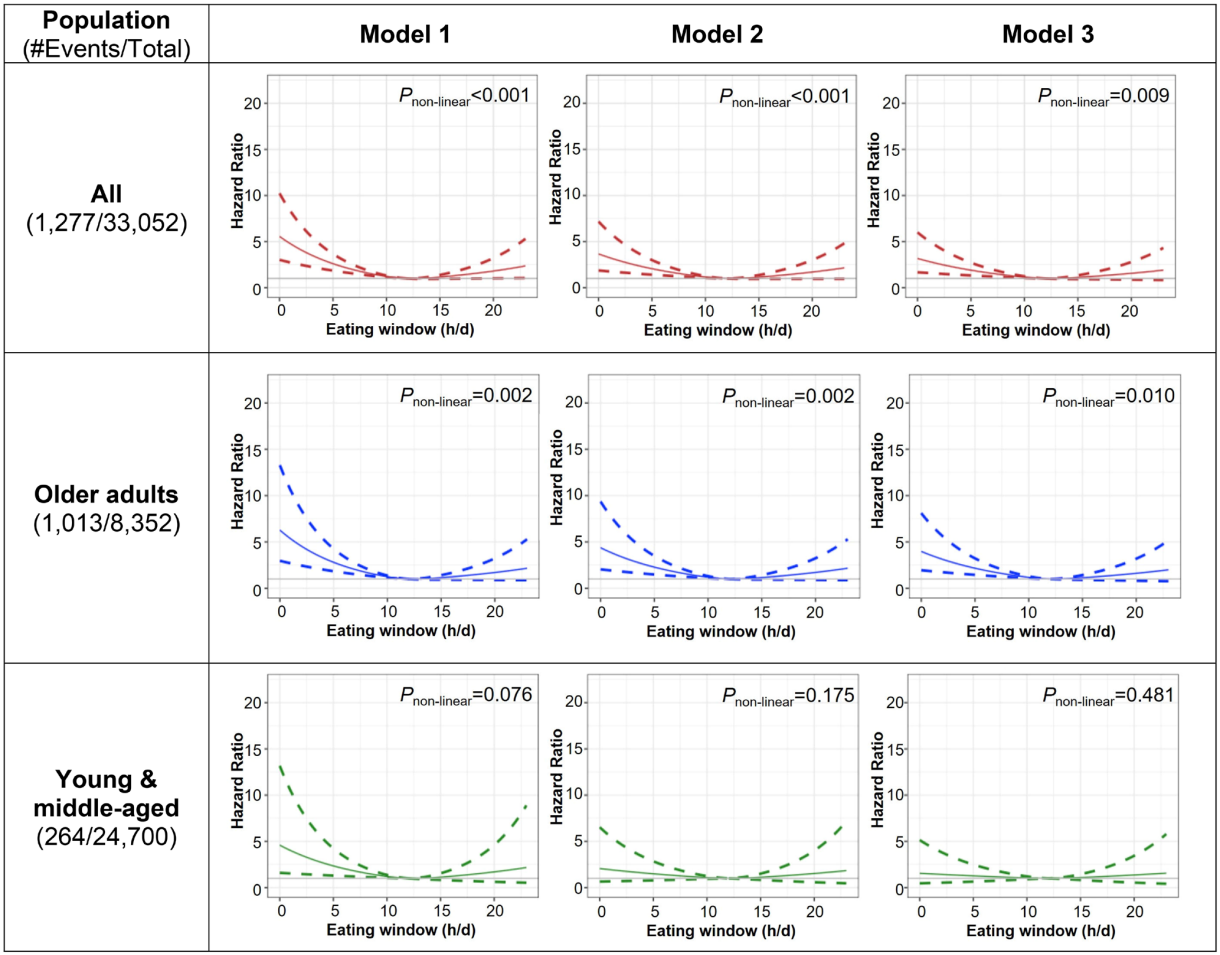
Survey‐Weighted Cox Regression with Restricted Cubic Splines (RCS) transformation for the association between eating window and cardiovascular mortality among US adults (*N* = 33,052): Overall and by age groups (Young and middle‐aged (20–64 years) and older (≥ 65 years) adults). Survey‐weighted multivariable Cox proportional hazards regression models were used to calculate hazard ratios (HRs; solid lines) and 95% confidence intervals (CIs; dashed lines). The shape of the associations was evaluated using RCS regression. Nonlinearity was assessed by comparing models: one with a linear term and another with cubic spline terms, using a likelihood ratio test to determine if there was a nonlinear relationship between the eating window and mortality. All hazard models were adjusted as follows: **Model 1**: Adjusted for age, age^2^, sex, race, and BMI categories. **Model 2**: Further adjusted for total calorie intake, diet quality, day of dietary intake, family income, alcohol intake, and smoking status. **Model 3**: Additionally adjusted for self‐reported health conditions, number of chronic conditions, moderate‐to‐vigorous physical activity, food security, self‐perceived body weight, attempts to lose weight in the past year, marital status, and education.

In the overall population, our RCS analysis identified a non‐linear U‐shaped relationship between eating window (treated as a continuous variable) and cardiovascular mortality after adjusting for all covariates (*p* = 0.009; Figure 4). The lowest risk was observed at an 11–12 h eating window in Model 3. Shorter eating windows (≤ 8 h/day) were associated with significantly higher cardiovascular mortality (HR ≥ 1.4) and 95% confidence intervals were consistently above 1. Although longer eating windows showed an upward trend in hazard ratios, the association was not statistically significant (Figure 4).

The subsequent stratified analysis revealed that the higher risk associated with shorter eating windows and cardiovascular mortality was particularly pronounced among older adults (Figure 4), men, and Whites (Figure S2). Specifically, in our fully adjusted RCS Model 3, shorter eating windows (≤ 8 h/day) were associated with HR ≥ 1.5 among older adults, HR ≥ 1.7 among men, and HR ≥ 1.5 among Whites, with *p* < 0.05 for non‐linear trends. Of note, longer eating windows (> 15.0 h) were significantly associated with higher cardiovascular mortality only in White participants in the RCS fully adjusted model 3, with HR rising to 1.5 or above (Figure S2). No similar association was found in other subgroups.

### 3.4 Cancer Mortality

In our RCS regression analysis, we observed an association between a shorter eating window and greater cancer mortality risk in the overall population (Figure S3), as well as in specific subgroups, specifically in younger/middle‐aged adults (Figure S3), women, and Whites (Figure S4), in the minimally adjusted Model 1. However, this association did not persist in the fully adjusted Model 3, except for a marginal association in women, where the lower limit of the confidence interval was slightly above 1 (Figure S4). Additionally, we observed no significant association between a longer eating window and cancer mortality, either overall or in our subgroup analyses.

## 4 Discussion

Using a nationally representative cohort of U.S. adults, our study demonstrated a non‐linear U‐shaped relationship between daily eating window and mortality risk. A moderate eating window of about 11–12 h/day was associated with the lowest mortality risks, independent of key demographic, dietary, and lifestyle factors. In contrast, shorter eating windows (≤ 8 h/day) were associated with a significantly higher risk of all‐cause mortality, showing a 30% or higher risk, particularly pronounced in older adults. Additionally, shorter eating windows were associated with over 50% higher cardiovascular mortality risk among older adults, men, and Whites. Furthermore, when treating eating window as a categorical variable, we observed a 25% higher risk of all‐cause mortality associated with the longest eating window category (≥ 15 h/day). This risk was particularly higher among Whites and was primarily driven by more than 50% higher risk of cardiovascular mortality.

Dietary approaches that limit eating to specific times of the day have recently gained attention for their potential health benefits. However, most existing evidence is based on animal studies, including those in *C. elegans* worms and fruit flies (de Cabo and Mattson 2019), and their impact on human longevity remains understudied. A growing body of intervention studies (Sun et al. 2024) has demonstrated that various time‐based diets can lead to mild weight loss (3%–8%) over 8–12 weeks, comparable to calorie restriction. These studies, which vary in design, duration, and population, have reported mixed effects on cardiometabolic risk factors, some showing improvements in blood pressure, cholesterol, and insulin (Sutton et al. 2018; Harvie et al. 2011), while others found no significant benefits (Lowe et al. 2020; Tinsley et al. 2019; Harvie et al. 2011). However, most of this evidence is limited to interventions over a short period of time, and their effects on longevity remain unclear.

### 4.1 Shorter Eating Window and Mortality Risk

Our findings reveal a consistent association between shorter eating windows (≤ 8 h/day) and greater all‐cause mortality across different demographics (including men, women, White, and Non‐White individuals). Notably, this relationship was particularly pronounced in older adults, with shorter eating windows significantly associating with over a 30% higher mortality risk, an association that was absent in younger adults. Further investigation into the causes of mortality unveiled that this higher risk was mainly due to cardiovascular mortality, with shorter eating windows associated with over 50% greater cardiovascular mortality among older individuals, men, and Whites. Our findings align with a recent study on U.S. adults, which reported an increase in all‐cause and cardiovascular mortality associated with nighttime fasting periods over 14 h daily using an earlier cycle of NHANES (Cheng et al. 2024). Another NHANES‐based study, limited to 10,561 adults aged over 60 from later NHANES cycles (2005–2018), also found that longer nighttime fasting periods were associated with higher CVD mortality risk (Zhang et al. 2024). Our study complements and extends these findings, as our larger sample directly compares younger versus older adults, incorporates a wider range of NHANES cycles (2003–2018), and evaluates mortality risks across the full spectrum of eating window durations. Additionally, we examined effect modification by age, sex, and race, and adjusted our models for relevant covariates such as diet quality, sleep duration, and weight‐loss dieting. Our broader scope and detailed analysis may have greater translational value. Also, by integrating these variables, we not only enhance the generalizability of our results but also deepen the understanding of how eating times affect diverse populations.

Mechanistically, longer fasting periods have been reported to affect the immune system, notably causing immune cell self‐destruction in the intestine (Nagai et al. 2019), and disrupt cortisol rhythms, a stress hormone (Hojlund et al. 2001). Aging also reduces resilience to metabolic stress due to shifts in circadian rhythms and increased cortisol levels (Moffat et al. 2020), weakening older adults' ability to handle stress and increasing frailty. Longer fasting periods (~15–16 h per day) might present an extra challenge to their physiological systems, raising the risk of adverse health effects. While longer fasting periods are generally considered to have minor risks, such as gastrointestinal and neurological changes, varying study results complicate safety evaluations (Varady et al. 2022) Some studies link long fasting periods with increased side effects, gallstone risk (Sichieri et al. 1991), and suggest that longer fasting hours, especially skipping breakfast, may correlate with higher mortality rates (Rong et al. 2019).

In our research, no link between shorter eating windows and lower mortality risk was observed in young and middle‐aged adults based on 24‐h dietary recall data. These observational findings suggest a more complex relationship and call for more comprehensive studies to reassess the health implications of such time‐based diets.

### 4.2 Longer Eating Window and Mortality Risk

Our study found that longer eating windows were associated with a 25% higher risk of all‐cause mortality, particularly among White individuals, with this association primarily driven by an increased risk of cardiovascular death. Previous studies have reported that longer eating windows, especially those continuing into the night, are associated with an increased risk of obesity, diabetes, and heart disease, particularly in younger adults (Yoshida et al. 2018). A recent NHANES‐based study (Li et al. 2024), using a single 24‐h dietary recall, found that adults with the longest eating windows (> 14 h/day) had higher all‐cause mortality in restricted cubic spline models. However, in their quartile‐based analyses, longer eating windows were associated with lower mortality risk. This discrepancy may stem from overly broad quartile categories used in their analysis (i.e., ≤ 11, 11–12.5, 12.6–14, and > 14 h/day), which may not adequately distinguish participants with narrower eating windows. Additionally, potentially relevant confounders such as diet quality, sleep duration, and adherence to weight‐loss diets were not accounted for in their models. Our study extends the existing literature by examining associations across narrower and biologically informed categories of eating windows, evaluating both all‐cause and cause‐specific mortality (cardiovascular and cancer mortality), and explicitly adjusting for overall diet quality (Healthy Eating Index) as well as other diet‐related factors (e.g., food security, perceived weight status). Moreover, we utilized two non‐consecutive 24‐h dietary recalls to enhance the representativeness and reliability of eating window estimates, thus providing a clearer assessment of the independent association between eating window and mortality. One potential mechanism involves late‐night eating which disrupts the body's natural insulin rhythm, that typically declines at night but is elevated by nocturnal eating (Yoshida et al. 2018). This misalignment with the body's circadian regulation has been shown to be associated with various metabolic disorders.

While we found significant associations between extended eating windows and higher risks of all‐cause and cardiovascular mortality, the same was not consistently observed for cancer mortality. This may be due to the lower incidence of cancer‐related deaths in our study compared to cardiovascular mortality, the leading cause of death among U.S. adults (Ahmad and Anderson 2024), limiting our statistical power to detect significant associations with cancer mortality. Additionally, research on dietary timing suggests that limiting eating windows to specific times of the day may reduce cancer risk by lowering oxidative stress and enhancing DNA repair (Tinkum et al. 2015). However, these findings are mostly from preclinical trials, with limited direct evidence in humans. Large‐scale studies are needed to clarify these associations and explore the mechanisms behind cancer mortality.

## 5 Strength and Limitations

The strength of our study lies in several key areas that enhance understanding of the association between eating windows and longevity. Using a nationally representative sample of U.S. adults, we identified a U‐shaped relationship between eating window and mortality, challenging linear associations. By applying restricted cubic splines to treat eating window as a continuous variable, we captured nuanced associations across a wide range of exposure. Importantly, our age‐stratified analysis highlights that the relationship between eating window and mortality varies by age, addressing a significant gap in current research. Additionally, leveraging a nationally representative dataset with high‐quality, time‐stamped dietary data improves the generalizability of our findings. Unlike Food Frequency Questionnaires, which do not capture intake timing, NHANES dietary recalls utilize the USDA 5‐step Automated Multiple‐Pass Method (AMPM), which explicitly records the timing of each reported food and beverage intake during step three of the interview, enabling precise determination of eating windows. The study's robustness is further supported by a follow‐up period of up to 17.1 years.

Despite the strengths of our study, our study has several limitations. First, although we used two 24‐h dietary recalls, a widely accepted tool for estimating diet in large‐scale studies (O'Hearn et al. 2022), the reliance on self‐reported data can lead to biases, including random or systematic errors (Willett 2012a). To reduce these biases, we included only individuals with *two* dietary recalls and used the average in our analyses. While averaging helps minimize random errors, it may not fully address systematic misreporting if certain groups consistently under‐ or overreport their intake. Second, 24‐h food recalls are prone to measurement errors due to daily dietary variation (Willett 2012a). To address this, we categorized recalls by day type (weekend/weekday) and included it as a covariate in our analysis. Additionally, we observed minimal day‐to‐day variability in mean eating window duration between the two dietary recalls collected at baseline (Day 1 vs. Day 2: 12.01 vs. 11.91 h, respectively; a difference of ~6 min), indicating relatively stable eating patterns within participants. Third, NHANES collects dietary data only at baseline, which may not fully capture dietary changes over time or seasonal variation. It is noteworthy that both of these limitations, that is, the lack of repeated dietary measurements and the inherent day‐to‐day variability in intake, owing to the study design of collecting just two recalls (one on a weekday and another on a weekend), are expected to attenuate the associations between predictors and outcome towards null (Willett 2012a, 2012b), as they reduce the precision in determining participants' eating window. The fact that our study showed significant associations between eating window and mortality despite random measurement error suggests robust associations, which are potentially underestimated. Future research incorporating repeated dietary assessments could further validate and refine these findings. Fourth, our observational design limits causal interpretations, as residual confounding cannot be ruled out despite adjusting for key demographic, socioeconomic, lifestyle, dietary, and health‐related variables. In addition, some of the adjusted confounders (e.g., diet quality, sleep duration, and physical activity levels) were assessed using self‐reported questionnaires, and unmeasured factors may still contribute to the observed associations, underscoring the need for future research to validate these findings.

## 6 Conclusion

Our findings suggest a U‐shaped relationship between eating window and mortality, with a moderate eating window of approximately 11–12 h/day associated with the lowest mortality risks. Deviations from this range, including shorter eating windows (< 8 h/day) and extended eating windows (≥ 15 h/day), were associated with higher mortality risks. However, we observed differing relationships between eating window and mortality across population subgroups, emphasizing the complexity of dietary timing and the need to account for individual and demographic differences when developing dietary recommendations. Further studies, including randomized controlled trials in diverse populations or large‐scale observational studies with repeated dietary assessments over time, are essential to confirm these findings, uncover underlying mechanisms, and inform evidence‐based public health guidelines.

## Author Contributions

S.F. designed and provided statistical consultation; Z.M. and H.G. analyzed the data; S.F. and Z.M. wrote the manuscript; A.B.N. and S.B.K., H.G. were involved in the interpretation of data and manuscript critical review; S.F. had primary responsibility for the final content; and all authors read and approved the final manuscript.

## Conflicts of Interest

The authors declare no conflicts of interest.

## Supporting information


**Figure S1:** acel70230‐sup‐0001‐FigureS1.docx.

**Table S1:** acel70230‐sup‐0002‐TableS1.docx.

## Data Availability

Data described in the manuscript, code book, and analytic code will be made publicly and freely available without restriction.
