# Supplementary material for: Association of Eating Window With Mortality Among US Adults: Insights From a Nationally Representative Study
Source: Aging Cell. 2025 Sep 13;24(11):e70230. doi: 10.1111/acel.70230 (PMC12610411; doi:10.1111/acel.70230)
Supplement: Supplementary file 1 — Figure S1: acel70230‐sup‐0001‐FigureS1.docx. [file ACEL-24-e70230-s001.docx]

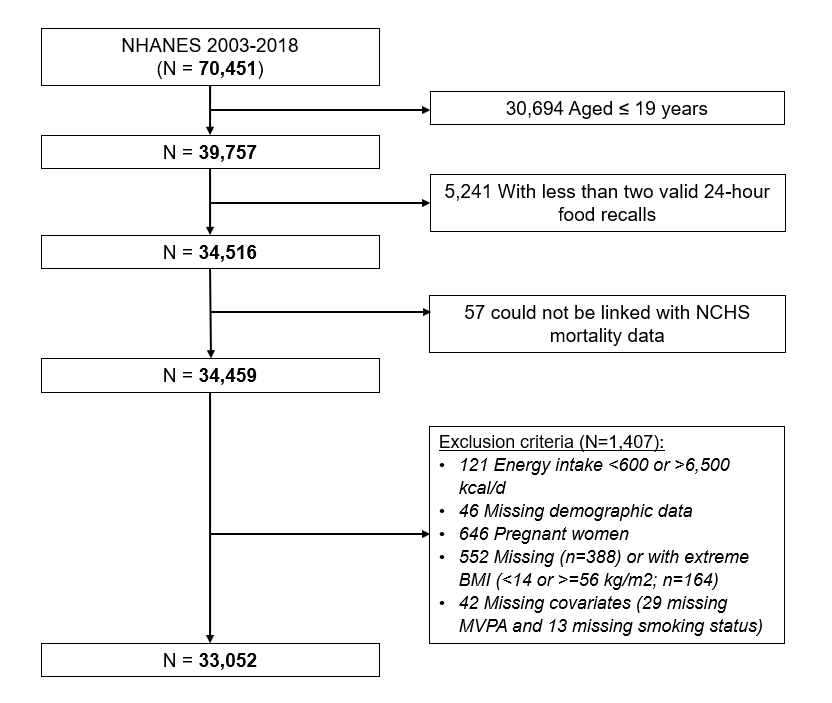


**eFigure 1** Participants flowchart.

| **Population** (#Events/Total) | **Model 1** | **Model 2** | **Model 3** |
| --- | --- | --- | --- |
| **Men**  (720/16,038) | 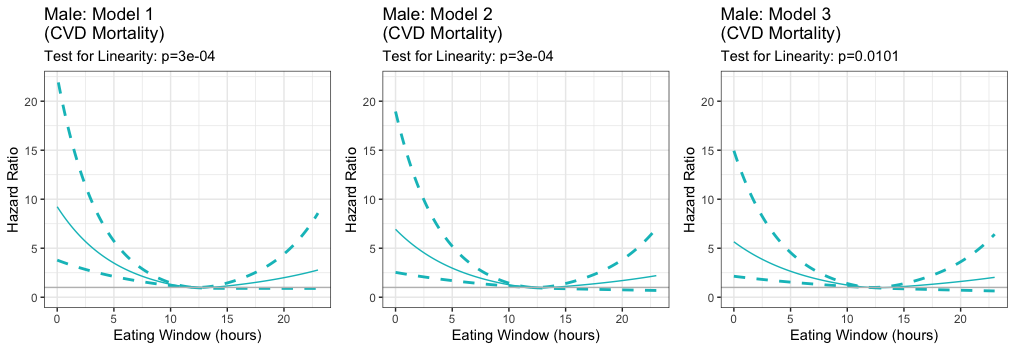  *P*_non-linear_=0.010  *P*_non-linear_<0.001  *P*_non-linear_<0.001 | | |
| **Women**  (557/17,014) | 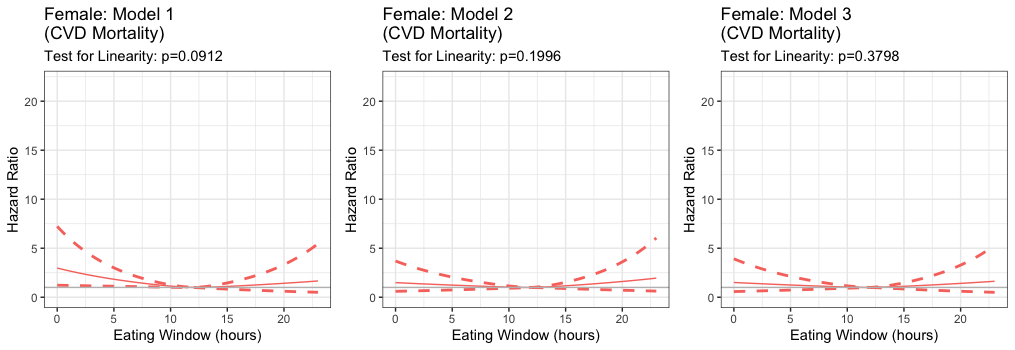  *P*_non-linear_=0.091  *P*_non-linear_=0.200  *P*_non-linear_=0.380 | | |
| **White**  (844/15,056) | 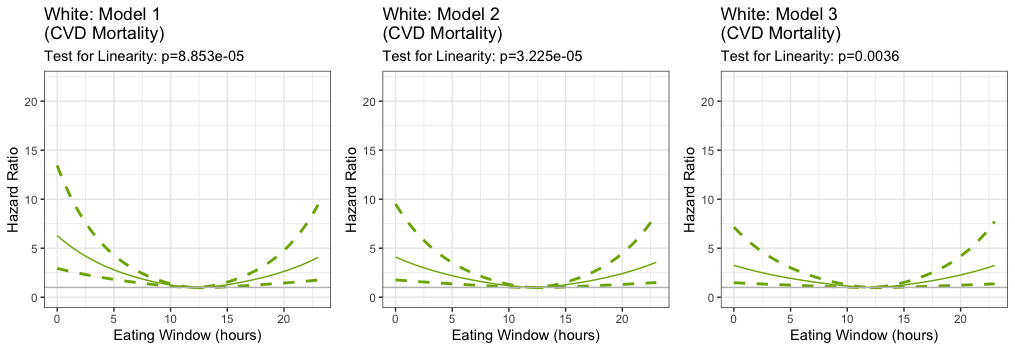  *P*_non-linear_=0.004  *P*_non-linear_<0.001  *P*_non-linear_<0.001 | | |
| **Non-White**  (433/17,996) | 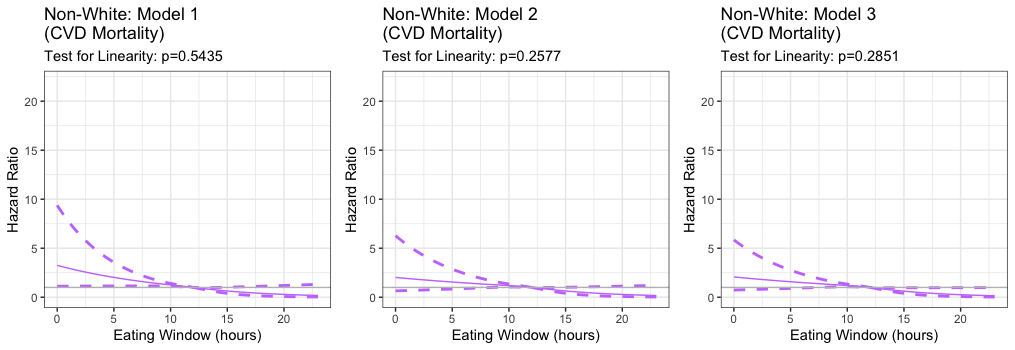  *P*_non-linear_=0.544  *P*_non-linear_=0.260  *P*_non-linear_=0.285 | | |

**eFigure 2** Survey-Weighted Cox Regression with Restricted Cubic Splines (RCS) transformation for the association between eating window and cardiovascular mortality among US adults (N = 33,052): sex (Men (n = 16,038) or Women (n = 17,014)) and race/ethnicity (White (n = 15,056) or Non-White (n = 17,996)) groups. Survey-weighted multivariable Cox proportional hazards regression models were used to calculate hazard ratios (HRs; solid lines) and 95% confidence intervals (CIs; dashed lines). The shape of the associations was evaluated using RCS regression. Nonlinearity was assessed by comparing models: one with a linear term and another with cubic spline terms, using a likelihood ratio test to determine if there was a nonlinear relationship between the eating window and mortality.

All hazard models were adjusted as follows:

**Model 1**: adjusted for age, age^2^, sex, race, and BMI categories.

**Model 2**: Further adjusted for total calorie intake, diet quality, day of dietary intake, family income, alcohol intake, and smoking status.

**Model 3**: Additionally adjusted for self-reported health conditions, number of chronic conditions, moderate-to-vigorous physical activity, food security, self-perceived body weight, attempts to lose weight in the past year, marital status, and education.

Stratified variable was not adjusted for in corresponding sex- or race-stratified models.

| **Population**  (#Events/Total) | **Model 1** | **Model 2** | **Model 3** |
| --- | --- | --- | --- |
| **All**  (989/33,052) | 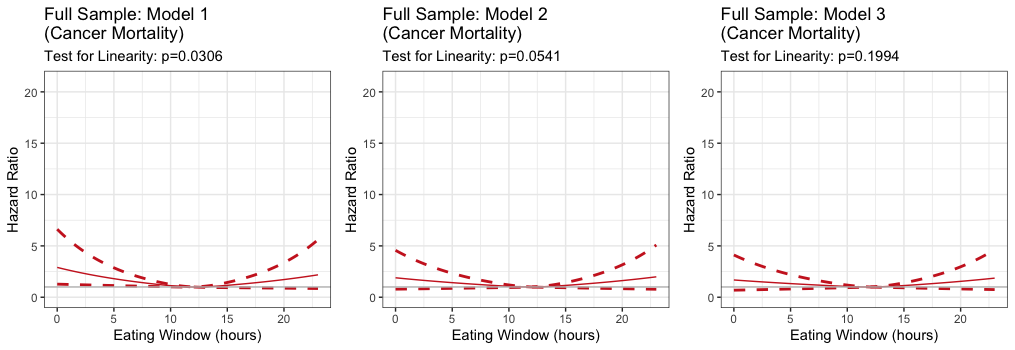  *P*_non-linear_=0.199  *P*_non-linear_=0.054  *P*_non-linear_=0.031 | | |
| **Older adults**  (652/8352) | 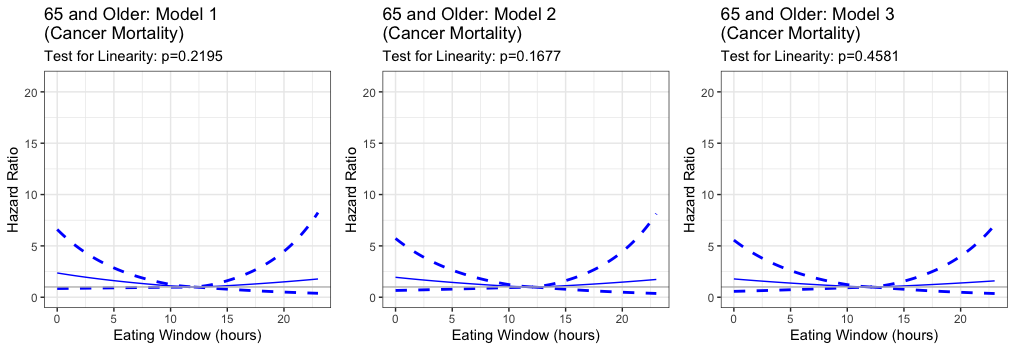  *P*_non-linear_=0.220  *P*_non-linear_=0.168  *P*_non-linear_=0.458 | | |
| **Young & middle-aged**  (337/24,700) | 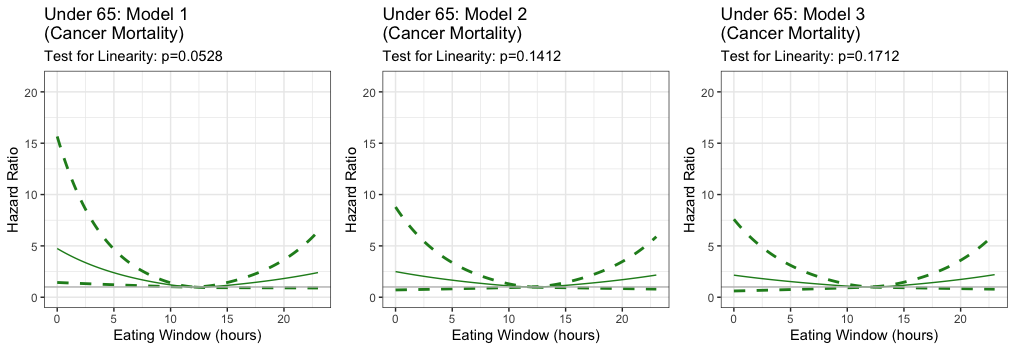  *P*_non-linear_=0.171  *P*_non-linear_=0.141  *P*_non-linear_=0.053 | | |

**eFigure 3** Survey-Weighted Cox Regression with Restricted Cubic Splines (RCS) transformation for the association between eating window and cancer mortality among US adults (N = 33,052): overall and by age groups (Young and middle-aged (20–64 years) and older (≥65 years) adults). Survey-weighted multivariable Cox proportional hazards regression models were used to calculate hazard ratios (HRs; solid lines) and 95% confidence intervals (CIs; dashed lines). The shape of the associations was evaluated using RCS regression. Nonlinearity was assessed by comparing models: one with a linear term and another with cubic spline terms, using a likelihood ratio test to determine if there was a nonlinear relationship between the eating window and mortality.

All hazard models were adjusted as follows:

**Model 1**: adjusted for age, age^2^, sex, race, and BMI categories.

**Model 2**: Further adjusted for total calorie intake, diet quality, day of dietary intake, family income, alcohol intake, and smoking status.

**Model 3**: Additionally adjusted for self-reported health conditions, number of chronic conditions, moderate-to-vigorous physical activity, food security, self-perceived body weight, attempts to lose weight in the past year, marital status, and education.

| **Population** (#Events/Total) | **Model 1** | **Model 2** | **Model 3** |
| --- | --- | --- | --- |
| **Men**  (591/16,038) | 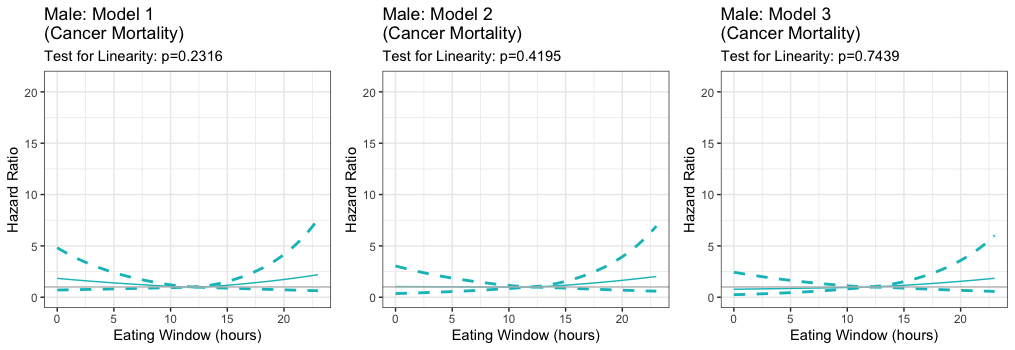  *P*_non-linear_=0.744  *P*_non-linear_=0.420  *P*_non-linear_=0.231 | | |
| **Women**  (398/17,014) | 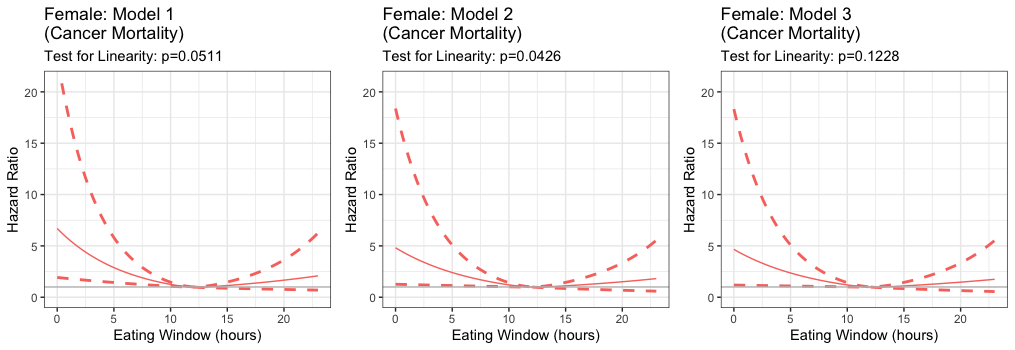  *P*_non-linear_=0.051  *P*_non-linear_=0.043  *P*_non-linear_=0.123 | | |
| **White**  (598/15,056) | 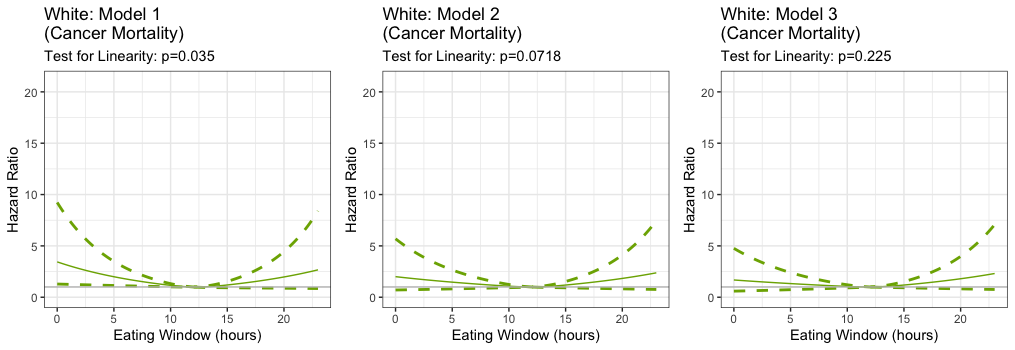  *P*_non-linear_=0.225  *P*_non-linear_=0.072  *P*_non-linear_=0.035 | | |
| **Non-White**  (391/17,996) | 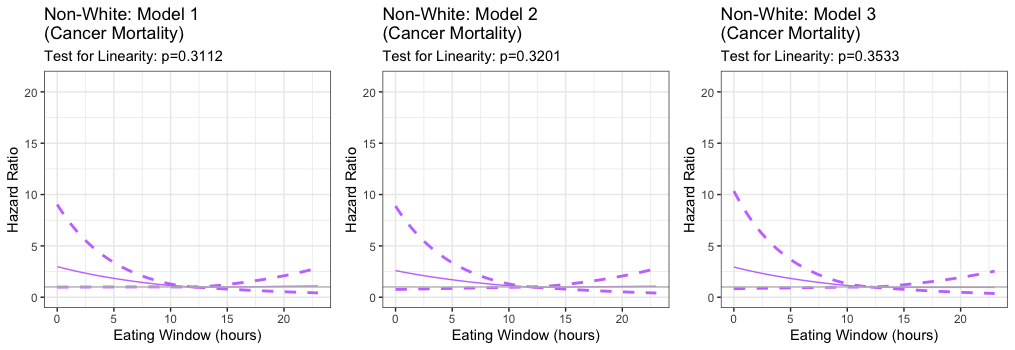  *P*_non-linear_=0.311  *P*_non-linear_=0.320  *P*_non-linear_=0.353 | | |

**eFigure 4** Survey-Weighted Cox Regression with Restricted Cubic Splines (RCS) transformation for the association between eating window and cancer mortality among US adults (N = 33,052): sex (Men (n = 16,038) or Women (n = 17,014)) and race/ethnicity (White (n = 15,056) or Non-White (n = 17,996)) groups. Survey-weighted multivariable Cox proportional hazards regression models were used to calculate hazard ratios (HRs; solid lines) and 95% confidence intervals (CIs; dashed lines). The shape of the associations was evaluated using RCS regression with three knots placed at the 25th, 50th, and 75th percentiles, selected based on the lowest Akaike Information Criterion. Nonlinearity was assessed by comparing models: one with a linear term and another with cubic spline terms, using a likelihood ratio test to determine if there was a nonlinear relationship between the eating window and mortality.

All hazard models were adjusted as follows:

**Model 1**: adjusted for age, age^2^, sex, race, and BMI categories.

**Model 2**: Further adjusted for total calorie intake, diet quality, day of dietary intake, family income, alcohol intake, and smoking status.

**Model 3**: Additionally adjusted for self-reported health conditions, number of chronic conditions, moderate-to-vigorous physical activity, food security, self-perceived body weight, attempts to lose weight in the past year, marital status, and education.

Stratified variable was not adjusted for in corresponding sex- or race-stratified models.
