# Supplementary material for: Association of Eating Window With Mortality Among US Adults: Insights From a Nationally Representative Study
Source: Aging Cell. 2025 Sep 13;24(11):e70230. doi: 10.1111/acel.70230 (PMC12610411; doi:10.1111/acel.70230)
Supplement: Supplementary file 2 — Table S1: acel70230‐sup‐0002‐TableS1.docx. [file ACEL-24-e70230-s002.docx]

**eTable 1**. Characteristics of participants included vs. excluded from the initial pool (N=39,757) of NHANES participants aged ≥20 years (2003-2018) **a**

|  | **Excluded** | **Included** | ***P* b** |
| --- | --- | --- | --- |
| **No. of participants** | **6,705** | **33,052** |  |
| Age, y | 42.4 ± 0.3 | 48.0 ± 0.2 | < 0.001 |
| Age categories, % |  |  | < 0.001 |
| 20-30 | 1878 (31.4%) | 5653 (18.6%) |  |
| 31-40 | 1337 (21.7%) | 5383 (17.6%) |  |
| 41-50 | 950 (17%) | 5627 (19.8%) |  |
| 51-60 | 818 (13.1%) | 5499 (19%) |  |
| 61-70 | 804 (7.9%) | 5507 (13.9%) |  |
| 71-80 | 804 (7.9%) | 4867 (10.4%) |  |
| 81 and above | 114 (0.9%) | 516 (0.8%) |  |
| Age group, % **c** |  |  | < 0.001 |
| Young and middle-aged | 5369 (87.2%) | 24700 (81.2%) |  |
| Older adults | 1336 (12.8%) | 8352 (18.8%) |  |
| Women, % | 3241 (50%) | 16038 (52.2%) | 0.009 |
| Race, % |  |  | < 0.001 |
| Hispanics | 1864 (17.2%) | 8031 (12.8%) |  |
| Non-Hispanic White | 2425 (57.9%) | 15056 (70%) |  |
| Non-Hispanic Black | 1621 (15%) | 6890 (10.6%) |  |
| Other/Multi-Racial | 795 (9.8%) | 3075 (6.6%) |  |
| Marital status, % |  |  | < 0.001 |
| Married | 3665 (56.5%) | 20079 (65.1%) |  |
| Single again | 1510 (18.9%) | 7367 (18.2%) |  |
| Never married | 1510 (24.6%) | 5606 (16.7%) |  |
| ≥College Graduate, % | 1078 (20.8%) | 7766 (30.1%) | < 0.001 |
| Income ≤$34k, % | 3352 (41.4%) | 14270 (32.6%) | < 0.001 |
| Never-smoked, % **d** | 3630 (53.4%) | 18084 (54.7%) | < 0.001 |
| Alcohol drinking, % | 5544 (86.1%) | 32459 (84.6%) | 0.008 |
| Very good health status, % | 1422 (25.7%) | 8622 (32.3%) | < 0.001 |
| CVD, % | 2239 (29.9%) | 12590 (33.3%) | < 0.001 |
| Cancer, % | 393 (5.6%) | 2862 (8.3%) | < 0.001 |
| Diabetes, % | 970 (10.9%) | 4930 (11.4%) | 0.419 |
| Number of Chronic diseases, 0-13 | 1.28 ± 0.03 | 1.48 ± 0.02 | < 0.001 |
| BMI (kg/m^2^), % **e** |  |  | 0.014 |
| Underweight (<18.5) | 119 (2.1%) | 493 (1.5%) |  |
| Normal (18.5-24.9) | 1819 (29.7%) | 8917 (28.4%) |  |
| Overweight (25.0-29.9) | 2007 (31.5%) | 11065 (33.1%) |  |
| Obese (≥30.0) | 2293 (36.6%) | 12577 (36.9%) |  |
| Lean mass, kg **f** | 53.4 ± 0.3 | 52.0 ± 0.2 | < 0.001 |
| Fat mass, kg **g** | 27.7 ± 0.4 | 28.3 ± 0.2 | < 0.001 |
| Sleep duration, h/d **h** | 7.13 ± 0.03 | 7.10 ± 0.01 | < 0.001 |
| MVPA, min/wk **i** | 165.3 ± 6.9 | 166.9 ± 2.8 | < 0.001 |
| Perceived weight status, % |  |  | < 0.001 |
| Overweight, | 3115 (50.4%) | 18183 (57.7%) |  |
| Underweight | 485 (6.7%) | 1652 (4.3%) |  |
| About the right weight | 3105 (42.9%) | 13217 (38%) |  |
| Weight loss attempt (past year), % | 1884 (30.9%) | 10572 (34.5%) | < 0.001 |
| Eating window, h/d | 11.5 ± 0.07 | 12.2 ± 0.02 | < 0.001 |
| Eating window, h/d, median [IQR] | 11.5 [9.5-13.0] | 12.0 [10.5-13.4] |  |
| Poor appetite or overeating, % **j** |  |  | 0.004 |
| Not at all | 4618 (75.7%) | 22657 (78.3%) |  |
| Several days | 895 (16.5%) | 4129 (14.1%) |  |
| More than half the days | 263 (4%) | 1267 (4.1%) |  |
| Nearly every day | 257 (3.8%) | 1223 (3.5%) |  |

*Abbreviations*: BMI, body mass index; CVD, cardiovascular disease (including hypertension, heart failure, coronary heart disease, or stroke); MVPA, Moderate to vigorous physical activity.

**a** Survey-weighted percentages and mean ± SE (or median [IQR] when indicated) were estimated using US population weights.

**b** *P* values from survey-weighted ANOVA for continuous variables and survey-weighted chi-square test for categorical variables.

**c** Age categories: Young and middle-aged (20–64 years) and older (≥65 years) adults.

**d** Smoking status (n = 39,735) was not assessed in 22 excluded participants.

**e** BMI (n = 39,290) was not assessed in 467 excluded participants.

**f** Fat mass (n = 17,884) assessed by DXA (QDR-4500A, Hologic Inc., Bedford, MA, USA) which was not available for NHANES cycles 2007-2010.

**g** Lean mass (n = 18,086) assessed by DXA (QDR-4500A, Hologic Inc., Bedford, MA, USA) which was not available for NHANES cycles 2007-2010.

**h** Self-reported usual sleep duration on weekdays (n = 35,206) was not available for NHANES cycles 2003-04.

**i** Physical activity (n = 39,713) was not assessed in 44 participants who did not have 2 valid dietary recalls.

**j** Appetite was assessed using the question 'Over the last 2 weeks, how often have you been bothered by poor appetite or overeating?' from NHANES cycles 2005-2018.

**eTable 2** Associations between daily eating window categories and all-cause mortality among U.S. adults aged ≥20 years after excluding participants who died within the first year of follow-up (N = 32,791) ^a^

| **Eating window categories**  **(hour/day)** | **Hazard Ratio** | **95% Confidence Intervals** | **P value** |
| --- | --- | --- | --- |
| < 8.00 | 1.27 | 1.01 - 1.59 | 0.04 |
| 8.00-9.99 | 1.09 | 0.93 - 1.27 | 0.29 |
| 10.00-10.99 | 1.11 | 0.95 - 1.30 | 0.20 |
| 11.00-11.99 | 1.03 | 0.90 - 1.17 | 0.70 |
| 12.00-12.99 |  | REF |  |
| 13.00-14.99 | 1.05 | 0.90 - 1.22 | 0.57 |
| ≥ 15.00 | 1.19 | 0.96 - 1.47 | 0.12 |

**a** Hazard ratios and 95% Confidence Intervals obtained from survey-weighted Cox regression model adjusted for age, age^2^, sex, race, BMI categories, total calorie intake, diet quality, day of dietary intake, family income, alcohol intake, smoking status, self-reported health conditions, number of chronic conditions, moderate-to-vigorous physical activity, food security, self-perceived body weight, attempts to lose weight in the past year, marital status, and education.

**eTable 3** Associations between daily eating window categories and all-cause mortality among U.S. Adults aged 40 and above (N =22615) ^a^

| **Eating window categories**  **(hour/day)** | **Hazard Ratio** | **95% Confidence Intervals** | **P value** |
| --- | --- | --- | --- |
| < 8.00 | 1.40 | 1.12 - 1.75 | 0.004 |
| 8.00-9.99 | 1.17 | 1.01 - 1.36 | 0.042 |
| 10.00-10.99 | 1.14 | 0.96 - 1.34 | 0.136 |
| 11.00-11.99 | 1.09 | 0.95 - 1.23 | 0.216 |
| 12.00-12.99 |  | REF |  |
| 13.00-14.99 | 1.08 | 0.93 - 1.25 | 0.305 |
| ≥ 15.00 | 1.27 | 1.03 - 1.58 | 0.028 |

**a** Hazard ratio and 95% Confidence Intervals obtained from survey-weighted Cox regression model adjusted for age, age^2^, sex, race, BMI, total calorie intake, diet quality, day of dietary intake, family income, alcohol intake, smoking status, self-reported health conditions, number of chronic conditions, moderate-to-vigorous physical activity, food security, self-perceived body weight, attempts to lose weight in the past year, marital status, and education. *Note: Individuals aged 20–39 years were excluded from this analysis due to potential influences of external factors, such as accidents on mortality outcomes*.

**eTable 4** Associations between daily eating window categories and all-cause mortality among U.S. adults aged ≥20 years adjusted for sleep duration (hours/day).

*Note: Sleep data were unavailable for NHANES 2003–2004 cycles (n = 29,200)* ^a^

| **Eating window categories**  **(hour/day)** | **Hazard Ratio** | **95% Confidence Intervals** | **P value** |
| --- | --- | --- | --- |
| < 8.00 | 1.40 | 1.11 - 1.78 | 0.006 |
| 8.00-9.99 | 1.13 | 0.95 - 1.35 | 0.166 |
| 10.00-10.99 | 1.20 | 1.01 - 1.43 | 0.036 |
| 11.00-11.99 | 1.07 | 0.93 - 1.23 | 0.325 |
| 12.00-12.99 |  | REF |  |
| 13.00-14.99 | 1.01 | 0.84 - 1.21 | 0.913 |
| ≥ 15.00 | 1.27 | 0.99 - 1.63 | 0.062 |

**a** Hazard ratios and 95% Confidence Intervals obtained from survey-weighted Cox regression model adjusted for age, age^2^, sex, race, BMI categories, sleep duration, total calorie intake, diet quality, day of dietary intake, family income, alcohol intake, smoking status, self-reported health conditions, number of chronic conditions, moderate-to-vigorous physical activity, food security, self-perceived body weight, attempts to lose weight in the past year, marital status, and education.

**eTable 5** Associations between daily eating window categories and all-cause mortality among U.S. adults aged ≥20 years adjusted for the employment status ^a^

| **Eating window categories**  **(hour/day)** | **Hazard Ratio** | **95% Confidence Intervals** | **P value** |
| --- | --- | --- | --- |
| < 8.00 | 1.32 | 1.05 - 1.65 | 0.017 |
| 8.00-9.99 | 1.13 | 0.97 - 1.31 | 0.131 |
| 10.00-10.99 | 1.17 | 0.99 - 1.38 | 0.058 |
| 11.00-11.99 | 1.06 | 0.94 - 1.21 | 0.337 |
| 12.00-12.99 |  | REF |  |
| 13.00-14.99 | 1.08 | 0.93 - 1.25 | 0.301 |
| ≥ 15.00 | 1.28 | 1.04 - 1.58 | 0.023 |

**a** Hazard ratios and 95% Confidence Intervals obtained from survey-weighted Cox regression model adjusted for age, age^2^, sex, race, BMI categories, employment status, total calorie intake, diet quality, day of dietary intake, family income, alcohol intake, smoking status, self-reported health conditions, number of chronic conditions, moderate-to-vigorous physical activity, food security, self-perceived body weight, attempts to lose weight in the past year, marital status, and education.

**eTable 6** Associations between daily eating window categories and all-cause mortality among U.S. adults aged ≥20 years after excluding <1st and >99th percentile of eating window (*n = 32,756*) ^a^

| **Eating window categories**  **(hour/day)** | **Hazard Ratio** | **95% Confidence Intervals** | **P value** |
| --- | --- | --- | --- |
| < 8.00 | 1.30 | 1.04 – 1.63 | 0.023 |
| 8.00-9.99 | 1.15 | 0.99 – 1.34 | 0.071 |
| 10.00-10.99 | 1.17 | 1.00 – 1.38 | 0.050 |
| 11.00-11.99 | 1.07 | 0.94 – 1.21 | 0.288 |
| 12.00-12.99 | REF | — | — |
| 13.00-14.99 | 1.07 | 0.93 – 1.23 | 0.366 |
| ≥ 15.00 | 1.27 | 1.02 – 1.57 | 0.032 |

**a** Hazard ratios and 95% Confidence Intervals obtained from survey-weighted Cox regression model adjusted for age, age^2^, sex, race, BMI categories, employment status, total calorie intake, diet quality, day of dietary intake, family income, alcohol intake, smoking status, self-reported health conditions, number of chronic conditions, moderate-to-vigorous physical activity, food security, self-perceived body weight, attempts to lose weight in the past year, marital status, and education.

**eTable 7** Correlation between daily eating window and lifestyle factors among U.S. adults aged ≥20 years ^a^

|  | **MVPA (min/week)** | **Sleep duration (hour/day)** | **Smoking status** | **Alcohol**  **drinking** | **General health condition** | **Food security** |
| --- | --- | --- | --- | --- | --- | --- |
| **Eating window** | 0.039 | -0.120 | 0.077 | 0.019 | -0.057 | -0.058 |
| **Sample size** | 33052 | 29200^b^ | 33052 | 33052 | 33052 | 33052 |

**a** Estimates are Pearson correlation coefficients

**b** Self-reported usual sleep duration on weekdays (N = 29,200) was not available for NHANES cycles 2003-04.
